# Fungal Endocarditis With Severe Vegetations of the Aortic Valve and Septic Emboli Secondary to Total Parenteral Nutrition

**DOI:** 10.7759/cureus.32357

**Published:** 2022-12-09

**Authors:** Andrii Labchuk, Mada Hamwi, Alice Han, Muhammad Khan, Arvey Stone

**Affiliations:** 1 Internal Medicine, Advocate Lutheran General Hospital, Park Ridge, USA; 2 Infectious Diseases, Advocate Lutheran General Hospital, Park Ridge, USA; 3 Cardiology, Advocate Lutheran General Hospital, Park Ridge, USA; 4 Critical Care Medicine, Advocate Lutheran General Hospital, Park Ridge, USA

**Keywords:** vegetations, medical icu, fungal endocarditis, transthoracic echocardiogram, total parenteral nutrition (tpn)

## Abstract

Fungal endocarditis is a rare but serious complication of fungemia. It is most commonly caused by *Candida* species. Risk factors include prosthetic heart valves, injection drug use, and indwelling central venous catheters. In comparison to bacterial endocarditis, fungal endocarditis is more commonly associated with arterial embolization, likely due to the larger size of vegetations. Unfortunately, diagnosis is often delayed, contributing to significant morbidity and mortality. Relapses are common, and extended treatment is often warranted. Antifungal agents and valve replacement are the recommended treatments. However, in-hospital mortality remains at 36%. For these reasons, it is critical to have a high index of suspicion and not delay appropriate therapy.

## Introduction

This case offers a possible presentation of fungal endocarditis and highlights the importance of early detection of fungemia in patients on total parenteral nutrition (TPN). Appropriate therapy should not be delayed in these patients. There are no clear or specific symptoms of fungal endocarditis that can challenge diagnosis or initiation of therapy. Despite readily available diagnostic tools and advanced antifungal therapy, fungal endocarditis is associated with poor prognosis. *Candida *and *Aspergillus *are the two prime etiologic agents of fungal endocarditis. *Candida *species account for approximately 50% of all cases of fungal endocarditis [1].

## Case presentation

A 67-year-old female with a medical history of pulmonary embolism, deep venous thrombosis (DVT), and small bowel obstruction that required bowel resection and the creation of a high-output ostomy complicated by enterocutaneous fistula presented to the emergency department for evaluation. The patient presented to the emergency department with abdominal pain, generalized weakness, and septic shock, with an elevated lactic at 3.6 mmol/L. Prior to the admission, the patient was residing in a skilled nursing facility. Table 1 shows the vital signs on admission.

**Table 1:** Vitals signs on admission

Parameter	Value
Blood pressure (mmHg)	80/29
Heart rate (beats per minute)	102
Respiratory rate (breaths per minute)	26
Oxygen saturation (%)	100% on 4 L of supplemental oxygen
Temperature (°F)	100.8

The patient was dependent on TPM through a dual lumen peripherally inserted central catheter (PICC) line. Four days before admission to the hospital, the patient developed a fever and altered mental status. Urine analysis was performed and was consistent with urinary tract infection. Table 2 shows the abnormal laboratory results on admission to the hospital.

**Table 2:** Pertinent abnormal laboratory results on admission to the hospital. WBC = white blood cell; HGB = hemoglobulin; HCT = hematocrit; PLT = platelet; AST = aspartate transaminase; SGOT = serum glutamic-oxaloacetic transaminase; ALT = alanine transaminase; SGPT = serum glutamic-pyruvic transaminase

Parameter	Value
WBC	11.0 K/µL
HGB	9.5 g/dL
HCT	32.8%
PLT	237 K/µL
Absolute neutrophil	9.6%
Chloride	113 mmol/L
CO_2_	16 mmol/L
Glucose	143 mg/dL
Creatinine	0.99 mg/dL
AST/SGOT	143 U/L
ALT/SGPT	142 U/L
Alkaline phosphatase	412 U/L
Albumin	1.8 g/dL
Lactic acid	3.6 mmol/L
Procalcitonin	2.12 ng/mL
High-sensitivity troponin	444 ng/L

**Figure 1:**
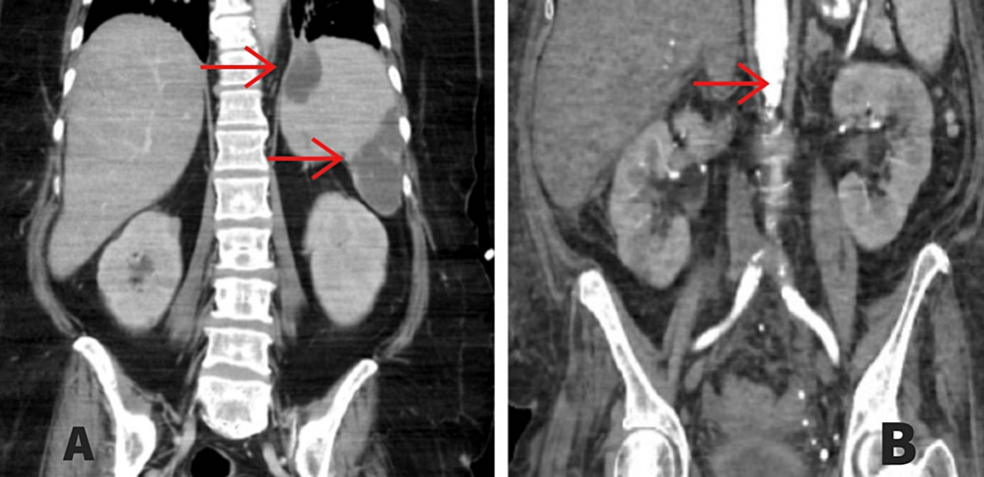
Computed tomography of the abdomen and pelvis. A: Septic emboli of the spleen. B: Septic emboli of the left kidney.

The patient was started on intravenous antibiotics; however, her fever did not improve and her condition progressively worsened. The patient was started on vasopressors and broad-spectrum antibiotics (Zosyn and vancomycin) in the emergency department. Computed tomography (CT) of the chest, abdomen, and pelvis showed multifocal areas of hypodense enhancement in the spleen and the renal inferior pole, concerning for emboli (Figure 1).

**Figure 2:**
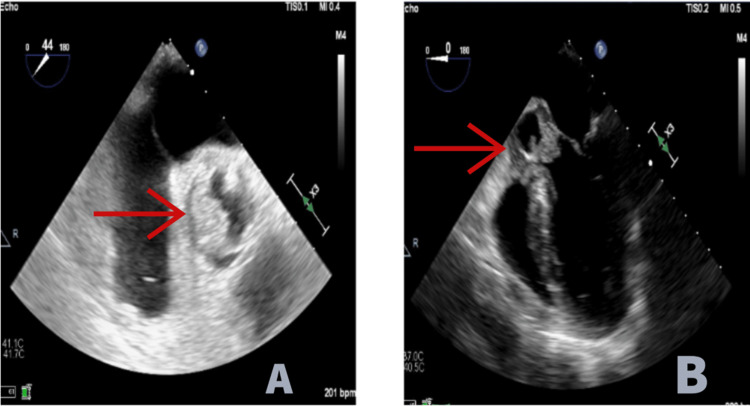
Vegetation of the aortic valve on TEE. A: Vegetations of the aortic valve at 44 degrees, short axis on TEE. B: Vegetation of the aortic valve at 0 degrees, mid-esophageal long axis on TEE. TEE = transesophageal echocardiogram

The patient was admitted to the medical intensive care unit for treatment of septic shock. Blood cultures were positive for *Staphylococcus epidermidis*, *Candida auris*, *Candida parapsilosis*, and *Candida tropicalis*. Infectious disease was consulted, and the patient was started on daptomycin, piperacillin/tazobactam, and micafungin. Her PICC line was removed, and the catheter tip was sent for culture. Ophthalmology was consulted, and dilated fundus examination showed chorioretinal lesions, consistent with fungal endophthalmitis. The initial transthoracic echocardiogram was unrevealing but limited due to the patient’s body habitus. She clinically progressed with worsening respiratory failure requiring intubation. A transesophageal echocardiogram was performed which showed large mobile vegetations of the aortic valve with severe aortic regurgitation (Figure 2).

A repeat blood culture showed persistent *Candida* species. Amphotericin B was added to her regimen given her worsening clinical status. Blood cultures were checked again and showed *Candida *species despite targeted treatment. Her hospital course was then complicated by a new onset of a cold and pale right lower extremity. Arterial doppler showed occlusion in the right distal common femoral artery, which was thought to be due to septic emboli. She was started on anticoagulation with a heparin drip; however, the treatment had to be discontinued due to progressive thrombocytopenia and anemia. The patient developed progressive lactic acidosis, multiorgan failure, and ultimately expired.

## Discussion

While TPN is often a necessity in critically ill patients, the complications can be devastating. Bloodstream infection remains one of the most common causes of mortality in these patients, particularly candidemia, as the lipids present in TPN may increase the virulence of fungi [1]. Some studies show that at least 5% of TPN recipients will develop candidemia. Of these patients, approximately 16% will develop infective endocarditis as a sequela [2]. The most effective way to decrease the risk of infection is to ensure proper hygiene and remove bloodstream catheters when they are no longer indicated. Prophylactic antifungals are not routinely recommended; however, they may be considered in patients with increased risk, particularly neutropenic patients.

Infective endocarditis should remain high on the differential in all patients on TPN who develop fever as it is the most common presenting symptom [3]. Imaging findings consistent with septic emboli should also clue clinicians into this diagnosis. Once the diagnosis of fungemia is established, central venous catheters should be removed as soon as possible. In addition, all patients should undergo an echocardiogram assessment to rule out endocarditis, in addition to ophthalmic consultation to assess for endophthalmitis [4]. In general, diagnosis of fungal endocarditis can be challenging because its clinical manifestation can be similar to bacterial endocarditis.

Fungal endocarditis has high mortality not just because of the nature of the disease but also due to its high association with an immunocompromised state [5]. Other risk factors are intravenous drug use, which in the past was thought to be the most common cause of fungal endocarditis, prostatic valves, indwelling central venous catheters, myelodysplastic syndrome, long-term use of corticosteroids, and immunosuppressive therapy after organ transplant [6]. As mentioned above, it is difficult to diagnose and differentiate fungal endocarditis due to an absence of a specific presentation. The most common presentation is a fever of unknown origin that is very common. In the described case, the patient was initially thought to have a urinary tract infection and was treated for that in the nursing facility with broad-spectrum antibiotics, but despite that, she was not improving. We want to highlight that in febrile patients with similar risk factors for fungal endocarditis, despite having suspicions regarding the source of the fever, it is important to keep fungal endocarditis high on the differential diagnosis. Fungal endocarditis should be considered in patients with peripheral embolization of the lower extremities, brain, and intra-abdominal organs. Other clinical symptoms could be weight loss, failure to thrive, splenomegaly, and petechial rash [7]. The challenging part of the diagnosis of fungal endocarditis is negative blood cultures in almost 50% of patients [8]. Transthoracic echocardiography is a readily available tool to evaluate heart valves for vegetation; however, transesophageal echocardiogram is a more sensitive and specific diagnostic method [9].

Complex multimodality treatment with antifungal medications and early surgical treatment with valve replacement and detriment of the vegetation is recommended due to the high morbidity and mortality of fungal endocarditis [10]. Combined antifungal therapy is superior to monotherapy. Chronic lifelong suppressive therapy with antifungal medication may be required [11].

Providers need to remember that embolic events are a frequent and life-threatening complication occurring in 13-49% of infective endocarditis patients [12]. Frequently, infective endocarditis is initiated by an endothelial injury that results in exposure of the subendothelial extracellular matrix that activates platelets and causes the formation of a fibrin-platelet clot. Subsequently, microorganisms in the blood adhere to the fibrin-platelet clot to initiate vegetation formation. These factors result in an invasive infection causing the release of cytokines. The bacteria-carrying particles cause systemic emboli primarily in left-sided infective endocarditis patients, while particles from the right side of the heart cause the majority of pulmonary embolisms [13].

The treatment of infective endocarditis should be guided by the causing pathogen and sensitivity to antibiotics. The duration of therapy in infective endocarditis must be sufficient to ensure the complete eradication of microorganisms within vegetation. In many cases, antibiotic therapy is empirical. During this time, empirical antimicrobial therapy is administered with the expectation that the regimen will be revised once a pathogen is defined until susceptibility results are obtained. The selection of an empiric regimen is usually broad and is based on factors related to patient characteristics, comorbidities, medical history, the severity of illness, prior antimicrobial exposure, microbiological findings, and epidemiological features [14]. Fungal endocarditis generally has a poorer prognosis compared to bacterial endocarditis. Despite treatment, mortality rates of 10% to 75% have been reported, stressing the importance of early diagnosis and treatment. Comorbidities prior to the diagnosis are often a cause of a worse prognosis. Complications of fungal endocarditis include systemic and central nervous system embolization, multiorgan failure, heart failure, and conduction block [15].

## Conclusions

Long-term TPN can be associated with the potential complications of fungemia which can lead to the development of infectious endocarditis. It is crucial to keep infectious endocarditis high on the differential diagnosis and initiate appropriate workup. Nowadays, fungal endocarditis is associated not only with drug use but also with various conditions such as immunosuppression due to therapy or bone marrow processes, chronic steroid use, prosthetic valves, indwelling catheter, and cardiac devices. A high index of suspicion for fungal endocarditis should be kept in patients in risk categories, especially in those presenting with prolonged fever of unknown origin. Complex and early treatment with combined antifungal medications and surgical intervention is still the only option to reduce morbidity and mortality from fungal endocarditis.

## References

[1] Badiee P, Amirghofran AA, Ghazi Nour M, Shafa M, Nemati MH (2014). Incidence and outcome of documented fungal endocarditis. Int Cardiovasc Res J.

[2] Kullberg BJ, Arendrup MC (2015). Invasive candidiasis. N Engl J Med.

[3] Sankar NP, Thakarar K, Rokas KE (2020). Candida infective endocarditis during the infectious diseases and substance use disorder syndemic: a six-year case series. Open Forum Infect Dis.

[4] Mermel LA (2000). Prevention of intravascular catheter-related infections. Ann Intern Med.

[5] Stratman RC, Martin CA, Rapp RP, Berger R, Magnuson B (2010). Candidemia incidence in recipients of parenteral nutrition. Nutr Clin Pract.

[6] Yuan SM (2016). Fungal endocarditis. Braz J Cardiovasc Surg.

[7] Cook RJ, Ashton RW, Aughenbaugh GL, Ryu JH (2005). Septic pulmonary embolism: presenting features and clinical course of 14 patients. Chest.

[8] Kaura A, Dworakowska D, Dworakowski R (2017). Infective endocarditis - Cinderella in cardiology. Kardiol Pol.

[9] Ellis M (1997). Fungal endocarditis. J Infect.

[10] Pappas PG, Kauffman CA, Andes DR (2016). Clinical practice guideline for the management of candidiasis: 2016 update by the Infectious Diseases Society of America. Clin Infect Dis.

[11] Lalani T, Chu VH, Park LP (2013). In-hospital and 1-year mortality in patients undergoing early surgery for prosthetic valve endocarditis. JAMA Intern Med.

[12] Berdejo J, Shibayama K, Harada K (2014). Evaluation of vegetation size and its relationship with embolism in infective endocarditis: a real-time 3-dimensional transesophageal echocardiography study. Circ Cardiovasc Imaging.

[13] Hu W, Wang X, Su G (2021). Infective endocarditis complicated by embolic events: pathogenesis and predictors. Clin Cardiol.

[14] Baddour LM, Wilson WR, Bayer AS (2015). Infective endocarditis in adults: diagnosis, antimicrobial therapy, and management of complications: a scientific statement for healthcare professionals from the American Heart Association. Circulation.

[15] Ojha N, Dhamoon AS (2022). Fungal Endocarditis. StatPearls Publishing.

